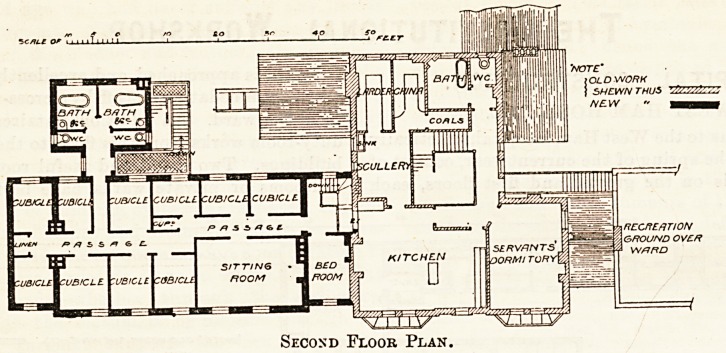# West Ham Hospital

**Published:** 1895-08-17

**Authors:** 


### THE INSTITUTIONAL WORKSHOP.

### HOSPITAL CONSTRUCTION.

### WEST HAM HOSPITAL.

The additions to the West Ham Hospital, practically completed in the spring of the current year, consist of two new wards on the ground and first floors, each the ward is approached, and excellently arranged sanitary accommodation cut off by a cross-ventilated lobby from the ward. An additional staircase opposite the duty-room works from this floor to the top of the new buildings. Two small and useful rooms, available as bedrooms or private wards, have been added on this

GROUND FLOOR PLAN.

accommodating twelve beds, with ten cubicles and a sitting-room for the nursing staff on the top floor. For some length of time it had been evident that further accommodation was needed, but lack of funds prevented any addition being possible until Mr. Passmore Edwards generously offered to bear the whole expense of building a new wing to the hospital, floor at the end of the men's ward in the old buildings.

The next floor repeats the ward (for 18 beds), duty-room, and sanitary accommodation in the new buildings, and adds two small private wards at the end of both the new and old wings, the total number of beds being now raised to 63. The top floor is disposed in dor-

FIRST FLOOR PLAN.

A glance at the plans published will show that in the main the new work, as far as the wards are concerned, is a repetition of one of the wings which originally existed, with the enlargement and improvement of sanitary arrangements connected with them. On the ground-floor a ward for twelve beds is provided, corresponding to the men's ward in the orig[illegible] institution, with a duty-room opening from the [illegible] by which mitory and other accommodation for the nursing staff. An external iron escape staircase is provided to the courtyard, with access from each of the three floors of the new building. The wards are warmed with open fire-places at both ends, and the arrangement of windows places one on either side of each bed in the usual way. The omission of a window between the end bed in each case and the end wall of the ward

Second Floor Plan.

seems an unfortunate oversight, when every facility for providing it occurred, and this is especially to be regretted as regards the beds next the doors leading to the sanitary blocks.

The steps in the corridor connecting the wards on the first floor were probably unavoidable in adding to the existing building, but they seem a blot upon plans marked otherwise by unusual care and completeness. The fittings are as follows:—Galton's open stoves with fresh air inlets; Cliff's enamelled earthenware baths, sinks, and slop hoppers; ventilators fixed at floor level and also window sill high; fire escape doors are fitted with Chubb's patent fire escape locks, iron outside staircase to each floor; the whole of the hot-water arrangements fitted by W. G. Cannon and Sons, 107, London Road, Southwark, S.E; and the contractors for the building are Messrs. Thomas White and Sons, Fairfield Road, Bow, E.

## Figures and Tables

**Figure f1:**
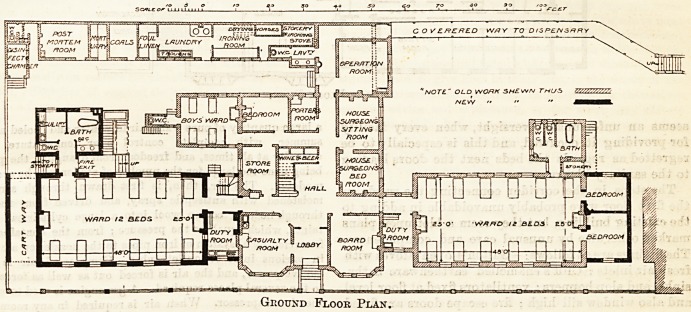


**Figure f2:**
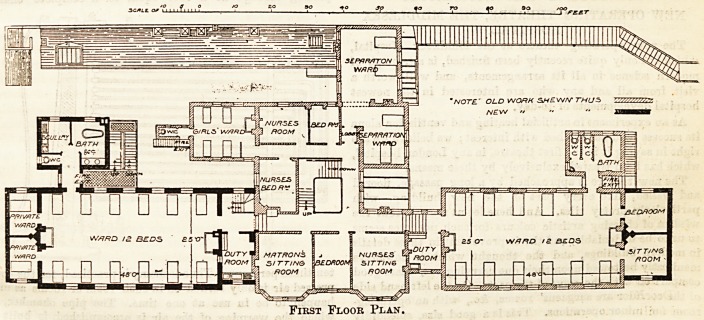


**Figure f3:**